# *hcapca*: Automated Hierarchical Clustering and Principal Component Analysis of Large Metabolomic Datasets in R

**DOI:** 10.3390/metabo10070297

**Published:** 2020-07-21

**Authors:** Shaurya Chanana, Chris S. Thomas, Fan Zhang, Scott R. Rajski, Tim S. Bugni

**Affiliations:** Pharmaceutical Sciences Division, School of Pharmacy, University of Wisconsin, Madison, WI 53705, USA; schanana@wisc.edu (S.C.); csthomas4@wisc.edu (C.S.T.); fzhang83@wisc.edu (F.Z.); scott.rajski@wisc.edu (S.R.R.)

**Keywords:** metabolites, genomics, PCA, HCA, dendrogram, variance, open source, LCMS

## Abstract

Microbial natural product discovery programs face two main challenges today: rapidly prioritizing strains for discovering new molecules and avoiding the rediscovery of already known molecules. Typically, these problems have been tackled using biological assays to identify promising strains and techniques that model variance in a dataset such as PCA to highlight novel chemistry. While these tools have shown successful outcomes in the past, datasets are becoming much larger and require a new approach. Since PCA models are dependent on the members of the group being modeled, large datasets with many members make it difficult to accurately model the variance in the data. Our tool, ***hcapca***, first groups strains based on the similarity of their chemical composition, and then applies PCA to the smaller sub-groups yielding more robust PCA models. This allows for scalable chemical comparisons among hundreds of strains with thousands of molecular features. As a proof of concept, we applied our open-source tool to a dataset with 1046 LCMS profiles of marine invertebrate associated bacteria and discovered three new analogs of an established anticancer agent from one promising strain.

## 1. Introduction

Natural product drug discovery programs continue to provide new and bio-medically relevant pharmacophores [1]. Among the potential sources of natural products, bacteria have proven to be a particularly prolific resource; for example, the genus *Streptomyces* is responsible for an unrivaled 80% of known actinomycete natural products [2]. Many natural products discovery programs rely heavily on collecting source organisms from diverse ecological niches in an attempt to harness the biological and chemical diversity stemming from these living systems. Given the time-span of a traditional natural product discovery pipeline [3,4,5], it is important to minimize the chemical redundancy and maximize chemical diversity in an environmental collection in order to minimize rediscovery. As such, tools to effectively survey sources of natural products prior to employing their chemistry for drug discovery are critical for effective discovery programs. Without effective tools, many downstream assay hits invariably result from similar or identical chemotypes, drastically increasing the number of resources required to discover new high-value leads. Although we have previously demonstrated that liquid chromatography mass spectroscopy (LCMS)-based metabolomics help to partly address this problem, we also found that there are fundamental limits to scaling these methods [6,7]. Specifically, there are no good tools to handle large LCMS-based untargeted metabolomics datasets that aligned with drug discovery goals. To meet this need we developed a tool called ***hcapca*** that enables rapid assessment of chemical diversity using low cost LCMS-based untargeted metabolomics.

As an alternative to LCMS techniques, Clark et al. recently demonstrated an excellent method to compare functional chemistry between closely related environmental isolates using in situ matrix-assisted laser desorption/ionization time-of-flight mass spectrometry (MALDI-TOF MS) based proteomics and metabolomics. They were able to discriminate between freshwater *Micromonospora* isolates that were more than 99% similar by 16S rRNA sequencing [8]. While MALDI clearly holds promise, many natural product discovery programs including ours, have chosen to utilize LCMS-based untargeted metabolomics techniques [3,9,10,11,12,13,14,15,16,17]. Compared to MALDI, reduced ion suppression [18], increased sensitivity, better metabolite coverage, and the ability to separate complex mixtures based on retention time all make LCMS a very attractive low-cost option for strain dereplication.

Given the appeal of LCMS, there have been a number of recent chemoinformatic advancements to better utilize its power, setting the stage for systems-level metabolomic investigations [19,20,21,22,23,24,25,26,27,28,29,30,31,32]. XCMS [26] and MZmine2 [32] are open source tools written to generate spectral tables that incorporate high-throughput peak detection and retention time correction. Tools such as GNPS [33] and MS-DIAL [24] aim to dereplicate molecular features using tandem MS data (MS2) while more recent techniques such as MASST [21] and Qemistree [34] enable one to search and classify those features based on publicly available molecular databases. However, many of these technologies, though amenable to large datasets, rely on MS2 data which provide rich sub-structure information but often focus only on the most intense ions. Although these innovative tools and techniques are extremely powerful, we believe that as a first step, ***hcapca*** can provide the necessary strain dereplication and help prioritize promising strains. Thereafter, optimization and additional scrutiny can be performed on the selected strains using MS2-based tools yielding even more chemical information.

While the tools used to identify strains with diverse chemistry are immensely important, the chemical diversity is also dependent on the environment where samples originate. New and diverse chemistry can be accessed by exploring non-traditional environmental niches such as caves and insect symbionts, making new sources for lead compounds available [35,36,37,38,39,40,41,42,43,44,45,46,47,48]. Although this increases the likelihood of finding new chemical entities, it is still difficult to identify these elusive molecules because a majority of the molecules produced are often shared, even across different genera. For example, Doroghazi et al. showed that related strains of actinobacteria—a phylum known for their prolific natural product potential [37,38]—shared 80% of their nonribosomal peptide synthetases (NRPSs) and 73% of their type II polyketide synthase (PKS) gene cluster families (GCFs) [49]. Additionally, Ziemert et al. and Jensen et al. showed that while there was a core set of metabolites within each of three species of *Salinospora*, unusual gene clusters were more random in occurrence [2,50]. In short, the microbial potential for chemical diversity does exist within organisms but is difficult to capture without specialized tools. ***hcapca*** employs principal component analysis (PCA), an unsupervised learning technique, which can highlight this hidden chemical diversity in an LCMS dataset. ***hcapca*** models variance in the data, collapses common metabolites, and highlights molecules that account for the greatest overall variance i.e., are likely to be “interesting”. Thus, ***hcapca*** enables users to access the diversity even within their large datasets to discover new chemistry.

Unsupervised techniques have been used in the past on metabolomics data [51,52,53,54,55,56,57,58,59] including hierarchical clustering analysis (HCA) [8,60]. Indeed, even within the realm of natural products discovery, we and others have successfully used PCA to identify novel chemical scaffolds [6,51,52,60,61,62,63,64,65,66,67]. However, we believe we are the first to rigorously integrate HCA with PCA. Thus, we present ***hcapca***—a general and highly effective algorithm designed to enable untargeted strain prioritization for drug discovery from large metabolomics datasets. As a proof of concept, we analyzed 1046 LCMS extracts from marine invertebrate associated bacteria resulting in 71,000+ molecular features. Using ***hcapca***, we rapidly organized this large dataset into 90 clusters. Upon examining one of the 90 clusters, we discovered three previously unknown analogs of lomaiviticin [68,69], an anticancer compound.

## 2. Results

### 2.1. PCA

Effective prioritization of samples for natural product drug discovery requires the identification of samples with unique chemistry. Unique or interesting chemistry can be identified by finding the sources of chemical variance amongst our samples. In our tool, we use PCA to model the variance in a dataset. PCA is a powerful algorithm that reorients data along the principal axes of variance in a dataset thus enabling the identification of interesting samples and subsequently, novel molecules [70,71]. PCA is agnostic of sample metadata such as species of the strains in the dataset or biological activity of the metabolites. This enables the discovery of chemically significant outliers [6,7,36,47,72,73,74] and important overarching patterns [17,70,71,75] even in datasets with little to no metadata i.e., datasets composed of samples from niche (often underexplored) environments.

**Figure 1:**
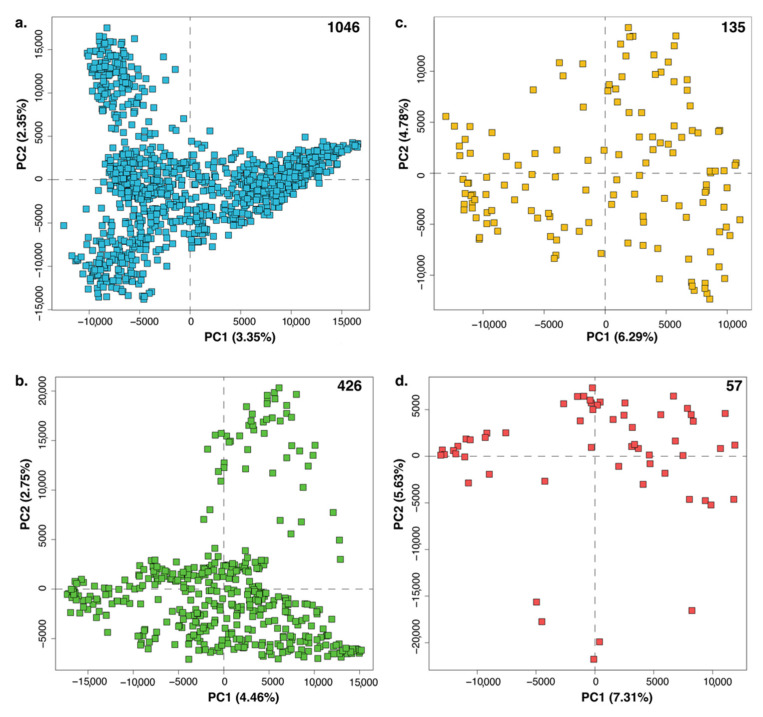
Subplots (**a**–**d**) show the PCA scores plot for four datasets. The number of samples in each dataset is shown in the top right corner of each plot. The total variance explained by a principal component (PC) is shown in parentheses next to the axis labels on each subplot. As the number of samples in a PCA decreases, the variance explained by each PC increases due to a combination of fewer samples and lesser overall variance in the dataset.

However, for very large datasets with more complex chemistry, PCA alone is insufficient to reveal clear trends. Ultimately, the model is dependent on the set of strains chosen for the dataset [7]. As shown in Figure 1, as we decrease the number of samples being modeled (numbers at the top right corner for subplots Figure 1a–d), trends become increasingly clearer. The amount of variance being explained by each principal component or PC (shown in parentheses on the axes of each subplot) also increases since there is less variation in the dataset. In Figure 1a, there are 1046 samples (and thus 1046 PCs) present and the first two PCs represent only 5.7% of the total variance in the dataset. For comparison, useful PCA models typically have far fewer PCs and an order of magnitude higher explained variance in PCs 1 and 2 than in the dataset being modeled [7,70,71,75].

### 2.2. HCA

**Figure 2:**
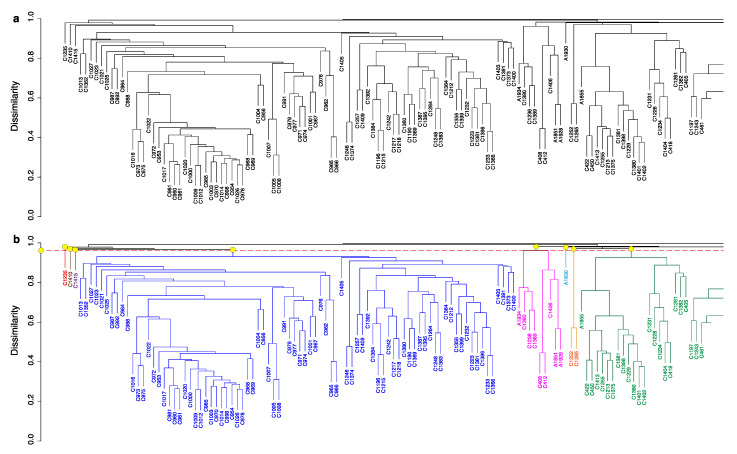
(**a**) Partial dendrogram generated from an HCA of all 1046 samples. The scale on the left denotes dissimilarity i.e., the closer to the bottom a pair of samples are, the more similar they are to each other. Only a small subset of the figure is shown for clarity; the original complete dendrogram may be found in the Supplementary Information as Figures S1 (linear display) and S3 (circular display). (**b**) Arbitrary dissimilarity cutoff choice of 0.95 results in eight different groups being formed. The groups have been colored accordingly. The eight groups have been colored as red, brown, grey, blue, magenta, teal, orange, and green. The yellow dots indicate the point at which the tree branch diverges to form each respective colored group of samples.

An overabundance of data in a single PCA model can be avoided by using HCA to split the dataset into smaller subgroups and then subjecting each of those subgroups to PCA. Analogous to clustering gene expression data [53], metabolite-based HCA assumes that samples with similar metabolic profiles are chemically related and should be grouped together. Performed alone, HCA is able to organize samples into a tree based on the similarity between microbial metabolomes. An example of such a tree, also called a dendrogram, is shown in Figure 2. The next step is to pick smaller clusters from this tree and subject each of them to PCA. However, while the human eye can pick out a few clusters or groups, the problem of choosing appropriate clusters quickly becomes intractable. Additionally, for very large datasets such as ours, dendrograms are extremely large and difficult to both visualize and interpret by human eyes alone. In fact, the dendrogram shown in Figure 2 is only a small portion (123 samples) of the tree representing all 1046 samples. Representations of the full tree can be found in Figures S1 and S3.

### 2.3. HCA and PCA in Combination

***hcapca*** solves the problem of sub-cluster generation and readily enables visualization of the sub-trees generated. Our aim is to create a tree based on the similarity between strains and then divide that tree into smaller sub-groups. Upon generating a tree such as Figure 2a, a decision must be made on how to divide this tree into smaller sub-groups. Typically, this is done by choosing a dissimilarity value on the *Y*-axis and drawing a line straight across the entire tree. The branches below the intersections of the straight line are considered separate sub-groups as shown in Figure 2b. Our next step would be to model the variance in these sub-groups using PCA. However, there are two problems that we must first solve. Firstly, there exist many values of dissimilarity cutoffs that would lead to groups with only one sample. Figure 2b shows how, if we chose a dissimilarity cutoff of 0.95, we are able to draw a line straight across (red dashed line) which intersects the tree branches at various points (colored yellow). These branches and the samples within are each treated as a separate group. Notice that the “groups” colored red, brown, purple (first three samples on the left in Figure 2b) and teal contain one sample each. PCA models are not possible for single samples. Secondly, the blue colored group in Figure 2b still contains 88 samples; far too many to allow for a robust PCA model. One simple solution is to regenerate a new tree from just the blue group and again choose an arbitrary dissimilarity cutoff to form sub-groups. While this solves our problem of having too many samples in a sub-group, our dissimilarity cutoff decision is still arbitrary and dependent on the choice of samples [7]. This is the same phenomenon we have observed with PCA—the choice of samples changes the PCA (or HCA) model completely [7].

**Figure 3:**
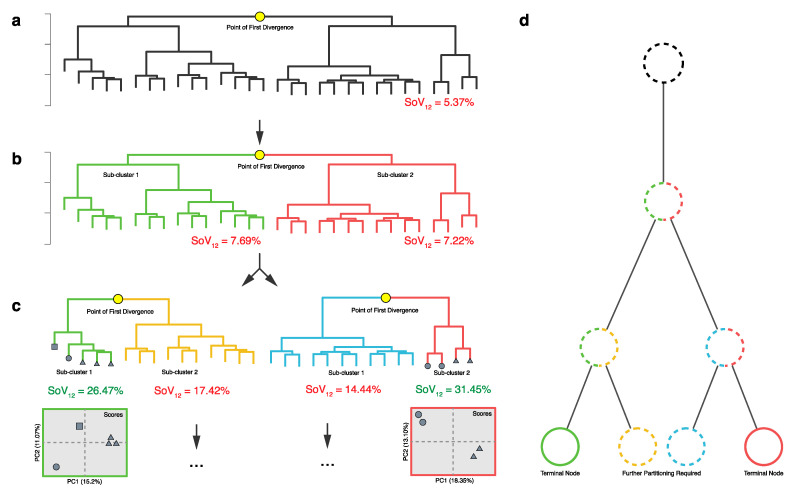
Scheme depicting ***hcapca*** logic. Note also that a small (35 sample) example of the walk through of ***hcapca*** processing and interactive visualization is depicted in Supplementary Information Figures S4–S13. (**a**) The first tree is partitioned into two smaller sub-clusters. (**b**) Since the $SoV_{12}$ for the two sub-clusters does not meet the cutoff value (25%), they are further split into smaller groups (**c**). The $SoV_{12}$ of the red and green clusters is more than the cutoff value and so their partitioning stops and PCA models are made (red/green squares). The green and blue sub-clusters have $SoV_{12}$s lower than the cutoff so they are split further as indicated by the ellipsis. (**d**) The overall structure of this schema results in a “tree-of-trees”. The circles represent the various nodes being formed and are colored as per the trees (from a, b, and c) that they represent. Dashed borders indicate nodes that need to be partitioned further while solid lines denote nodes that can no longer be split.

Herein lies the innovation of ***hcapca***; instead of choosing an arbitrary dissimilarity cutoff value for making sub-groups, we can use the variance explained by the PCs (from a PCA model) to decide the cutoff for us. First, as before, a distance matrix of all the samples is generated and a large tree is made (Figure 3a). Next, the tree is partitioned at the point where it first branches (point of first divergence in Figure 3a–c), and the sum of the variance is explained by the first two PCs ($SoV_{12}\;$) is simultaneously calculated from PCA models of each sub-cluster. If the $SoV_{12}$ is smaller than the preset cutoff value (25%), the cluster is re-partitioned (this condition is denoted in red color below each cluster). If the $SoV_{12}$ is greater than or equal to the preset cutoff value, the cluster is no longer partitioned (this condition is denoted in green color below each cluster) and a PCA model of the samples in that sub-cluster is calculated (the squares at the bottom of Figure 3c).

Using explained variance as a user-set cutoff for determining cluster composition and R Shiny [76,77,78] based interactive visualizations, ***hcapca*** thus helps to visualize the breakdown of the large tree into smaller and smaller sub-trees based on the chemical similarity within the data. 

In effect, the overall process yields a large “tree-of-trees” where each node represents a smaller tree. This representation is shown in Figure 3d where the overall tree is drawn parallel to each corresponding part of Figure 3a–c, and colored based on the splitting. Nodes that can be further partitioned are indicated by dashed borders and ones that will no longer be split are indicated by solid lines. In Figure 3d, two of the nodes reached the cutoff threshold (solid red and green borders); the other two did not and consequently were partitioned further (dashed blue and yellow circles). Thus, by combining HCA with PCA and recursive partitioning of the tree, we can obtain small, chemically similar sample groupings that yield more informative PCA models.

### 2.4. Identification of Novel Chemistry

**Figure 4:**
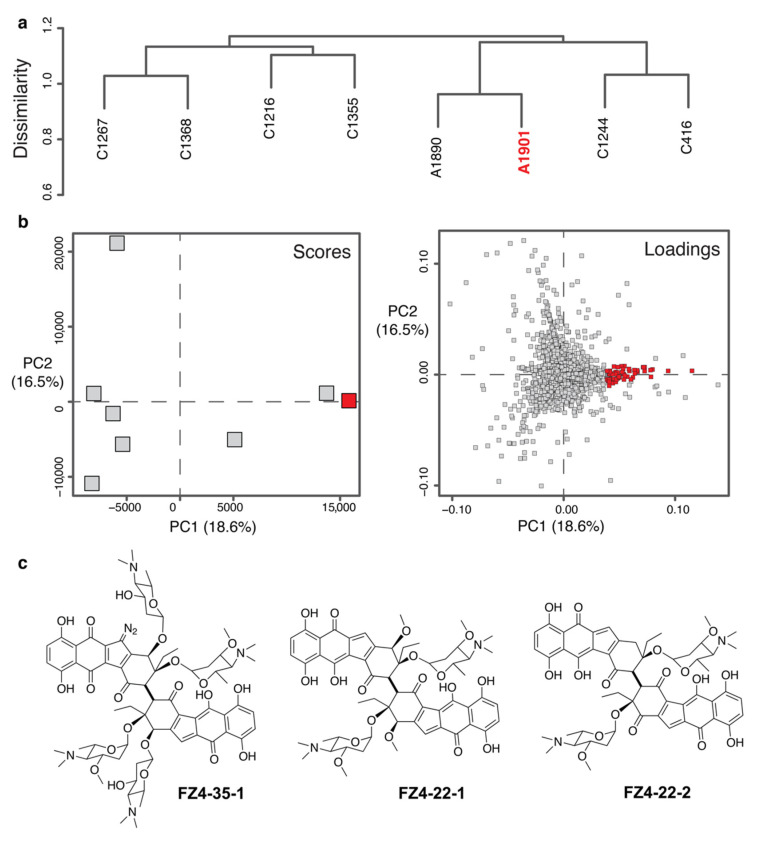
A1901 was identified from the PCA of node ‘fj’ shown in Figure S2. (**a**) The dendrogram of the node ‘fj’ contains eight strains in total. (**b**) PCA scores and loadings plots of the node containing A1901 with red squares highlighting the strain and its corresponding metabolites respectively are also shown. (**c**) Structures of the new lomaiviticin congeners.

It is important to reiterate our hypothesis that “outliers” in metabolomic data are more likely to be novel. Using PCA to model the chemical variance in a dataset, identify promising strains and their metabolites, and thereby discover novel molecules is a credible approach backed up by both our group’s own work [6,7,17,36,47,48,73,74], as well as by genomic studies [49,50] done by other groups. The addition of HCA serves to broaden the scope to allow large scale analyses and offer a more robust method of identifying promising strains. To demonstrate and utilize this algorithm for strain prioritization and drug discovery, we examined PCA models of the terminal nodes more closely and identified bacterium WMMA1901 (henceforth A1901) as a possible producer of novel chemistry (Figure 4). Figure S2 shows the location of the node in the overall tree. The HCA for this node contained eight samples (Figure 4a) and the PCA model for the node showed that the strain harbored interesting chemistry (Figure 4b). The highlighted red square on the Scores plot shows how A1901 is pulled out in the PCA. Since the scores and loadings plots are algebraically and geometrically related, the red squares on the loadings plot highlight the metabolites of interest that likely originate from strain A1901. Subsequent traditional isolation and purification by HPLC and structure elucidation by NMR revealed three new molecules (Figure 4c). Compounds **FZ4-23-1**, **FZ4-22-1**, and **FZ4-22-2**, are analogs of lomaiviticin, a class of anticancer compounds [68,69].

**Figure 5:**
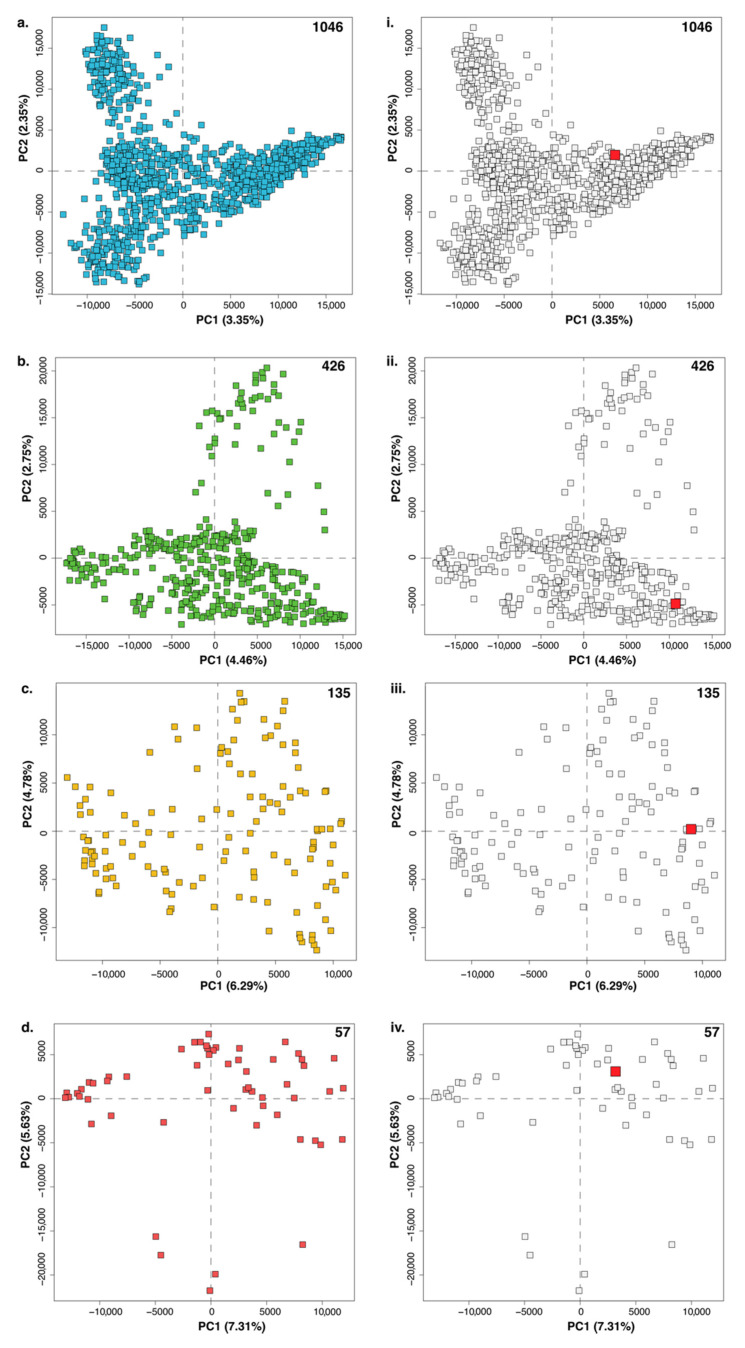
(**a**–**d**) represent the PCA models for the nodes mw, yq, ss, and bm from Figure S2, respectively. Sub-plots (**i**–**iv**) correspond to (**a**–**d**), respectively, highlighting the position of A1901 using a red dot while de-emphasizing other points in the plot by making them grey. Without the utilization of ***hcapca***, the discovery of the new anticancer compounds would not have been possible.

To further illustrate the utility of our tool, we highlighted the position of A1901 (see Figure 5 below) in each of the four PCA plots shown previously in Figure 1. It is immediately apparent that, without ***hcapca***, neither A1901 nor its unique metabolites would be discovered so expediently.

## 3. Materials and Methods 

Previously generated LCMS data from our library were used for this analysis. The library itself was created through isolation of bacteria from sponge and ascidian specimens, cultivation in solid or liquid media, extraction using solvents, and finally a UPLC-HRMS analysis to generate LCMS profiles. Please refer to our previous publications for the details [6,7,17] and to Supplementary Information Table S1 for all media employed during fermentations.

### 3.1. Generation of Spectral Intensity Tables

#### 3.1.1. Profile Analysis

Bruker Compass ProfileAnalysis 2.3 (Billerica, MA, USA). Find Molecular Features was applied to LCMS data under these parameters: S/N threshold, 5; correlation coefficient threshold, 0.7; minimum compound length, 10; smoothing width, 1. The LCMS datasets were evaluated in a time range from 2 to 14 min and in a mass range from *m/z* 150 to 1500. Advanced bucketing was employed using ∆RT = 0.33 min and ∆*m/z* = 4 ppm as parameters.

#### 3.1.2. MZmine2

We generated mass lists (detected ions) for each scan using Mass Detection (cutoff of 1E3), detected chromatograms using Chromatogram Builder (∆*m/z* = 4 ppm, ∆RT = 0.33 min), and separated individual peaks using the Chromatogram Deconvolution module (using ADAP module: an S/N threshold of 5, peak duration of 0 to 0.33 min, and RT wavelet range of 0 to 10 min). Isotopes were removed using the Isotopic peak grouper module and alignment was performed using both RANSAC and Join aligners. Finally, the data was exported to a CSV file and separated into 4 parts as specified in the Data format section below. 

#### 3.1.3. Data Format

The script expects data in four different files: Analyses.dat contains the sample names separated by new line characters, Variables_m.dat contains the *m/z* values separated by spaces all on one line, Variables_t.dat contain retention time values separated by spaces all on one line, and Table.dat contains the spectral intensity values separated by spaces with one line for each sample. Each row of Table.dat contains the intensity for the corresponding analysis name in the same order as the Variables_m and Variables_t values. It is important to realize that, even though the script was written to accommodate LCMS data, ***hcapca*** can be adapted to other kinds of data. For example, if a user does not have data for both *m/z* and retention time, they can fill one of the tables with zeroes and it has no bearing on the shape of the tree, placement of the nodes, etc.; i.e., the results are subject to contextual interpretation. Examples of the table formatting are available at—https://github.com/chanana/hcapca#table-format.

#### 3.1.4. Hierarchical Clustering Analysis (HCA)

Using the spectral intensity table (Table.dat), a distance matrix was first calculated using the Euclidean distance between each sample along its vector of *m/z*-rt values.

(1) $$d\left(p,q\right)=\sqrt{\overset{n}{\underset{i=1}{\sum }}{\left(q_{i}-p_{i}\right)}^{2}}$$

where, d is the distance between points $p=\left(p_{1},p_{2},\ldots \;,\;p_{n}\right)$ and $q=\left(q_{1},q_{2},\ldots \;,\;q_{n}\right)$. Here p can be thought of a sample in ${\mathbb{R}}^{n}$ with the *m/z*-rt pair $p_{i}$ representing a coordinate in each dimension.

Then, clustering was performed by employing the unweighted pair group method using the arithmetic mean (UPGMA) using correlation as a distance measure. Subsequently, the dataset was partitioned into the first two clusters that formed. This process was repeated on the resulting two clusters until the user-specified variance cutoff was met.

#### 3.1.5. Principal Component Analysis (PCA)

Once the entire tree had been built using the procedure described above, the clusters at the ends of the trees (the terminal nodes) were subjected to PCA. The data at each terminal node was Pareto scaled [79] and mean subtracted before PCA was performed. To demonstrate visually what the entire tree looks like prior to the construction of more refined elements of the dendrogram we refer readers to Figures S1 and S3 of the Supporting Information.

#### 3.1.6. Displaying Results

The results of ***hcapca*** data processing were displayed using our custom-made Shiny app [76], an interactive web design package written for the R programming language [78]. This app can be accessed once the code is run based on the instructions located at https://github.com/chanana/hcapca. The app runs on the local system of the user and is specific to each analysis the user performs.

#### 3.1.7. Source Code and Instructions

https://github.com/chanana/hcapca

## 4. Conclusions

Unique molecules within a large dataset generally have an increased likelihood of being new or novel. We have demonstrated that ***hcapca*** is able to leverage this property by being able to differentiate the A1901 metabolome from all other samples in its subgroup leading to the discovery of the lomaiviticin analogs. Our tool is open source, written in R [78], and encompasses all steps from the HCA, partitioning of the tree, and subsequent PCA of the terminal nodes requiring only a table of LCMS spectral intensities and a cutoff variance as input. Importantly, ***hcapca*** is available as a Docker image allowing it to be run on Windows, macOS, and Linux (Ubuntu) operating systems. Both proprietary and open-source software such as MZmine2 [32] can be used for upstream processing of raw LCMS files to generate the spectral tables required as the input; with appropriately formatted data tables (please refer to the Github repository or the Data Format section of the Methods for details). The installation instructions, as well as the entire source code for ***hcapca*** is available to the public for free at https://github.com/chanana/hcapca. As exemplified here with the discovery of new secondary metabolites from A1901, ***hcapca*** represents an important technology enabling the rapid identification of unique data points from very large datasets and is virtually unlimited in its potential applications to assorted scientific fields. 

## Supplementary Materials

The following are available online at https://www.mdpi.com/2218-1989/10/7/297/s1, Figure S1: Full dendrogram of all 1046 samples obtained by HCA. The figure’s actual size is 192 × 9″ making it extremely difficult to visualize at a detailed level, Figure S2: Depiction of the tree-of-trees i.e., the processing of all samples via ***hcapca***, Figure S3: The large tree (of 1046 samples seen in Figure S1) represented as a circular dendrogram using iToL, Figure S4: Screen shot of first step in analyzing HCA and PCA results following ***hcapca*** of a dataset, Figure S5: Screen shot of tree tab (with instructions) of ***hcapca*** results, Figure S6: Example of how to view a given dendrogram for a specific node viewable by drop-down menu, Figure S7: Screen shot of window in which PCA tab showing results of overall tree can be used to view the node and its “parent” nodes, Figure S8: How to navigate the PCA tab of results from ***hcapca*** processing of the example dataset provided, Figure S9: Demonstration of how the Scores plot (from Figure S8 steps) gets drawn and can be used to identify samples with greatest variance, Figure S10: Screen shot of the Loadings plot and how metabolite samples within a specific microbial producer vary from each other, Figure S11: Depiction of how ***hcapca*** leads to the plot showing individual and cumulative variances for all principal components in a given sample, Figure S12: Screen shot of first “log off” page enabling one to exit the ***hcapca*** application, Figure S13: Screen shot of page indicating completion of exit from hcapca application, Table S1: Comprehensive listing of all media ingredients used to generate data herein for the 1046 sample set.
