# Supplementary material for: hcapca: Automated Hierarchical Clustering and Principal Component Analysis of Large Metabolomic Datasets in R
Source: Metabolites. 2020 Jul 21;10(7):297. doi: 10.3390/metabo10070297 (PMC7407629; doi:10.3390/metabo10070297)
Supplement: Supplementary file 1 [file metabolites-10-00297-s001.pdf]

Supplementary Information for

hcapca: Automated Hierarchical Clustering and Principal Component Analysis of Large Metabolomic Datasets in R

Shaurya Chanana,[†] Chris S. Thomas,[†] Fan Zhang,[†] Scott R. Rajski,[†] and Tim S. Bugni[*†]

[†]Pharmaceutical Sciences Division, School of Pharmacy, University of Wisconsin, Madison, Wisconsin 53705, United States

*e-mail: tim.bugni@wisc.edu

Table of contents

Supporting Item	Description of contents	Page/s
**Figure S1**	Full dendrogram of all 1046 samples obtained by HCA. The figure's actual size is 192 x 9" making it extremely difficult to visualize at a detailed level.	**S3**
**Figure S2**	Depiction of processing of the 1046 samples by ***hcapca***.	**S4**
**Figure S3**	The large tree (of 1046 samples seen in **Fig. S1**) represented as a circular dendrogram.	**S5**
**Figures S4–S13**	A walk-through of online *hcapca* processing of data set available via GitHub accessible script. Example provided at GitHub is of 35 sample dataset derived from marine microbe extracts.	**S6–S15**
**Figure S4**	Screen shot of first step in analyzing HCA and PCA results following *hcapca* analysis of large data set.	**S6**
**Figure S5**	Screen shot of tree tab (with instructions) of *hcapca* results	**S7**
**Figure S6**	Example of how to view a given dendrogram for a specific node viewable by drop-down menu	**S8**
**Figure S7**	Screen shot of window in which PCA tab showing results of overall tree can be used to view the node and its "parent" nodes	**S9**
**Figure S8**	How to navigate the PCA tab of results from *hcapca* processing of the example dataset provided	**S10**
**Figure S9**	Demonstration of how the Scores plot (from Figure S8 steps) gets drawn and can be used to get identify samples with greatest variance	**S11**
**Figure S10**	Screen shot of the Loadings plot and how metabolite samples within a specific microbial producer vary from each other	**S12**
**Figure S11**	Depiction of how *hcapca* leads to plot showing individual and cumulative variances for all principal components in a given sample	**S13**
**Figure S12**	Screen shot of first "log off" page enabling one to exit the *hcapca* application	**S14**
**Figure S13**	Screen shot of page indicating completion of exit from *hcapca* application	**S15**
**Table S1**	Comprehensive listing of all media ingredients used to generate data herein for the 1046 sample set	**S16–17**
**References**	References used in this supplementary information file	**S17–20**

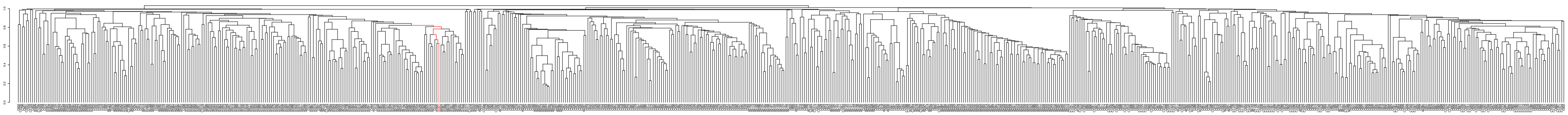

Figure S1. *Dendrogram of all 1046 samples obtained by HCA. The actual size of the figure is 192 x 9” making it extremely difficult to visualize in any significant level of detail or resolution.*

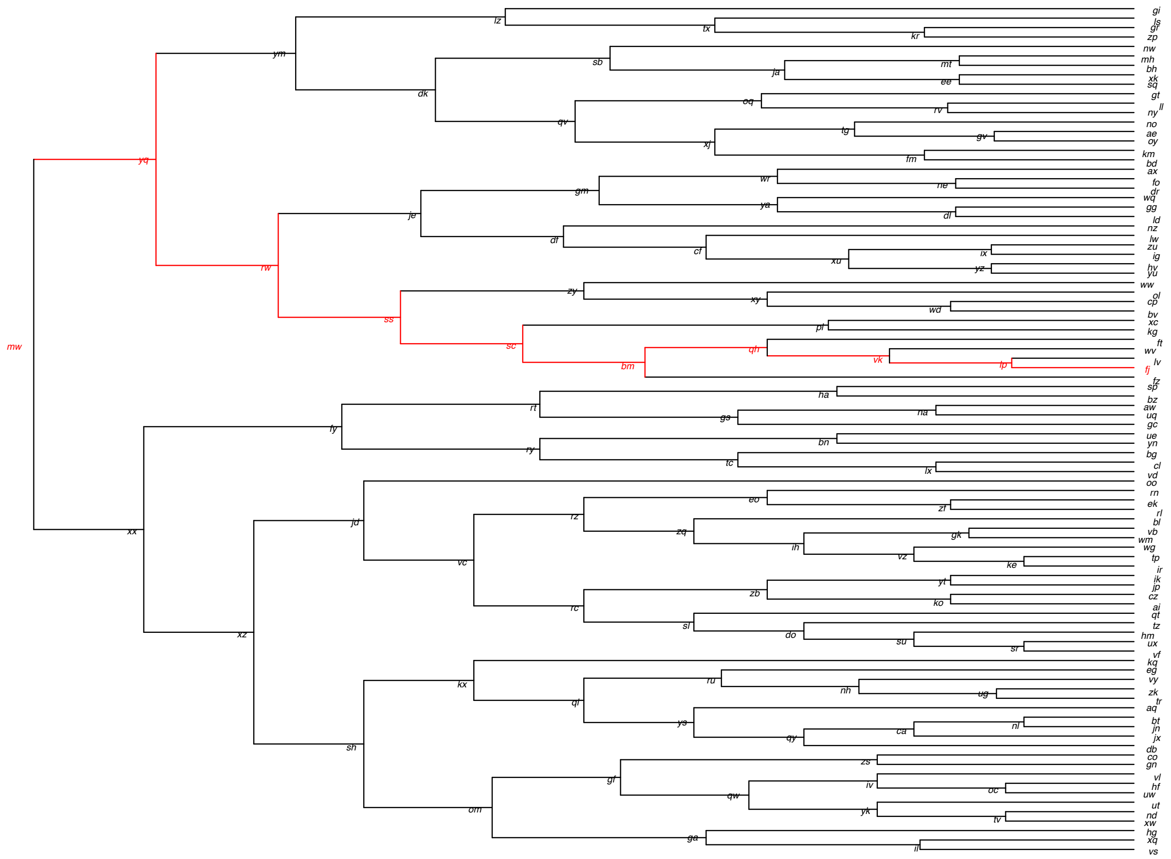

Figure S2. *The tree shown above represents the processing of the 1046 samples by hcapca. Each labeled point is called a node. The root node (mw) contains all 1046 samples. The red path highlighted represents the location of strain A1901 as the clusters became smaller and smaller before finally ending up in the terminal node (fj) with 8 samples. The path is as follows: mw → rw → ss → sc → bm → qh → vk → lp → fj.*

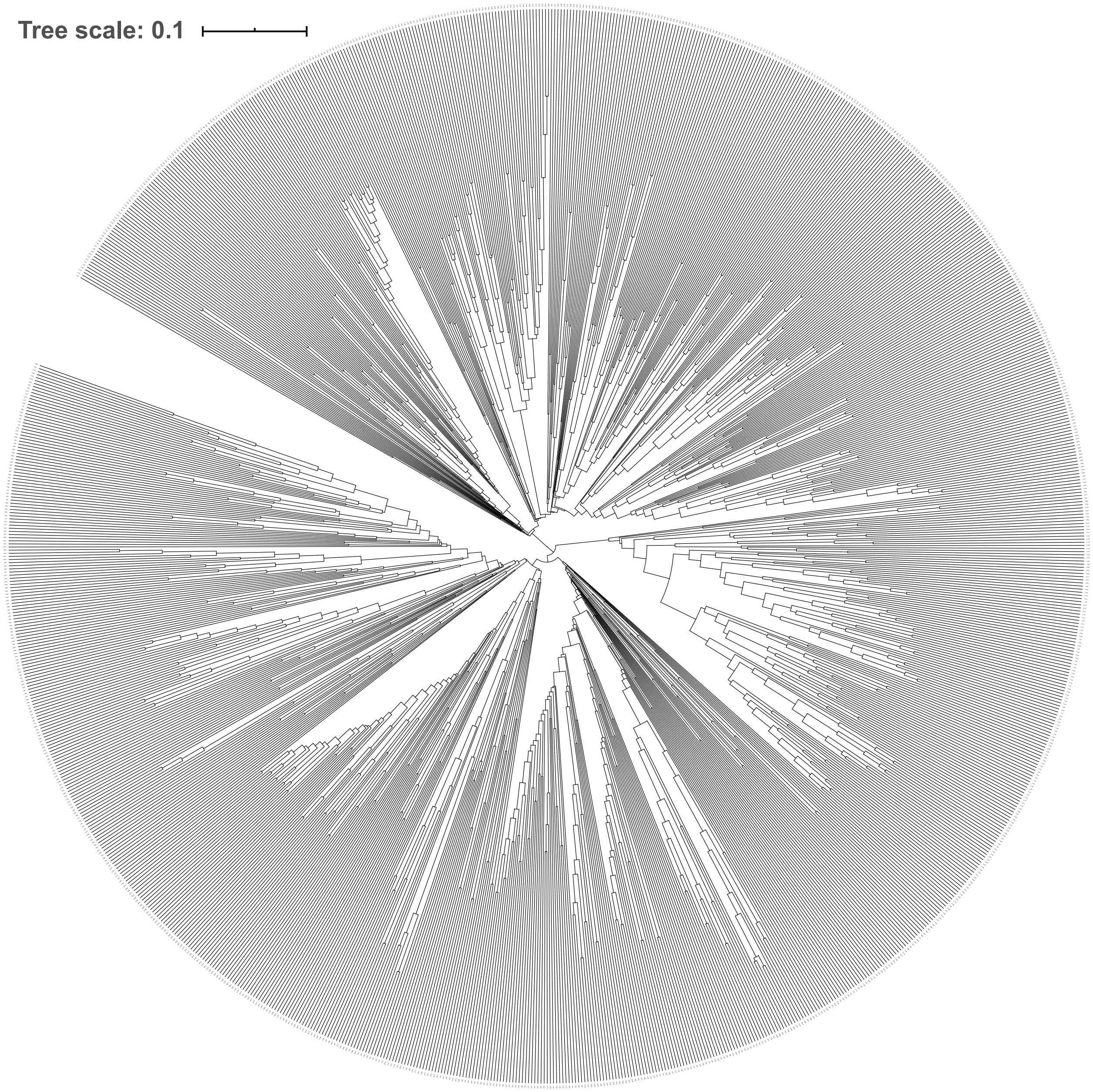

Figure S3. *The large tree represented as a circular dendrogram. The tree was generated using the Interactive Tree of Life v4 [1]*

1. *Letunic, I.; Bork, P. Interactive Tree of Life (iTOL) v4: recent updates and new developments. Nucleic Acids Res 2019, 47, W256–W259, doi:10.1093/nar/gkz239)*

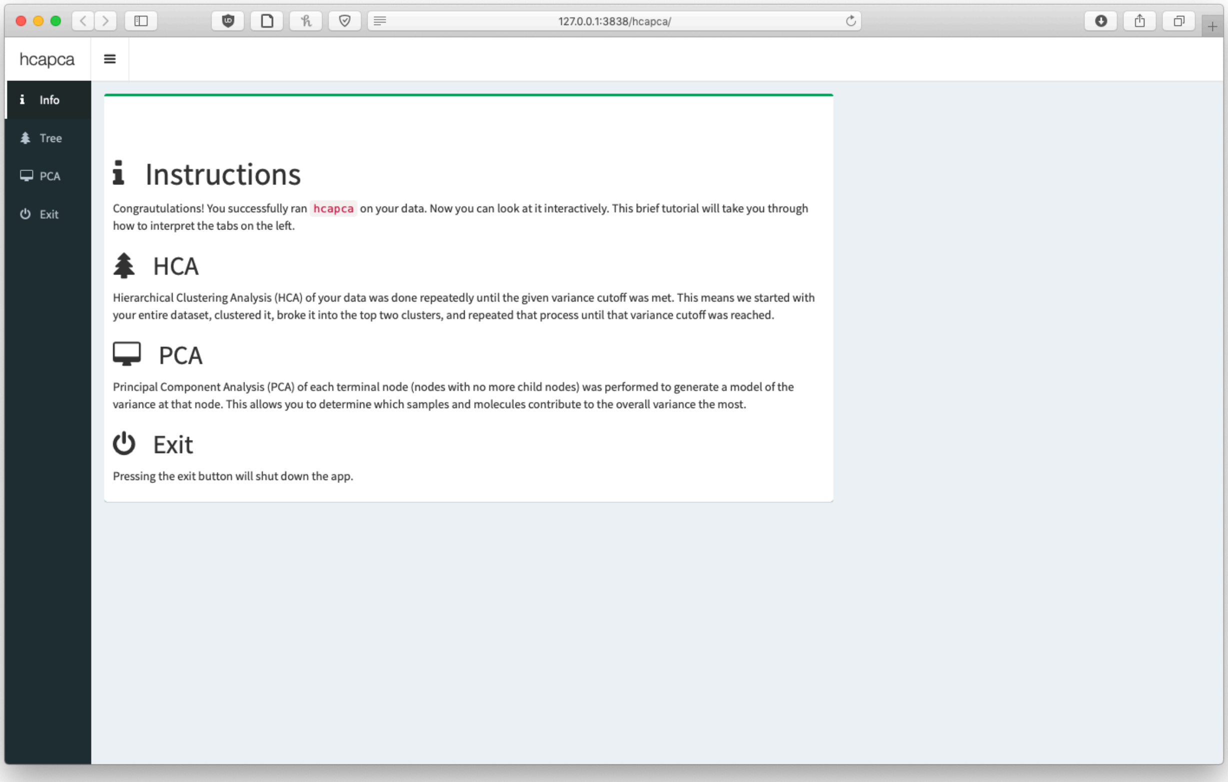

Figure S4. *The first page of the results. It contains basic instructions on what was done to the data and how to navigate the results.*

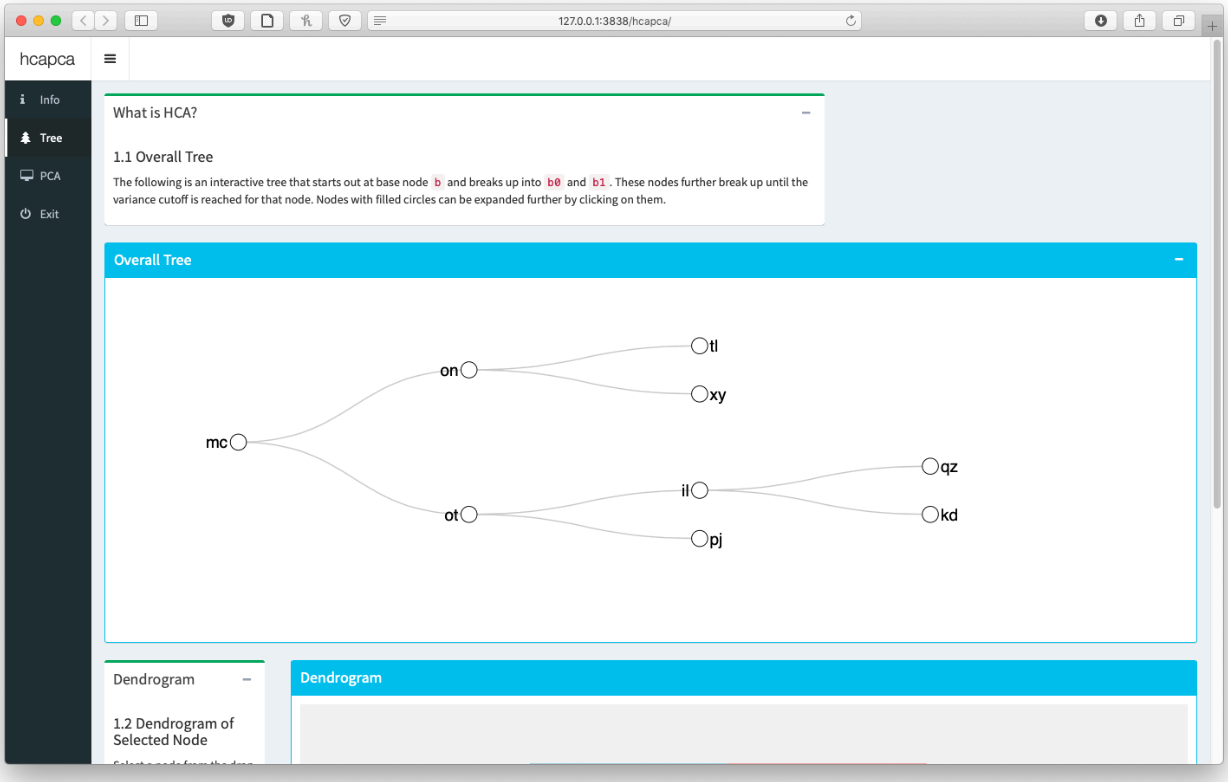

Figure S5. *The "Tree" tab of the results. It instructs the user on what hierarchical clustering analysis (HCA) is and how to navigate the tree shown in the blue box labeled "Overall Tree" below.*

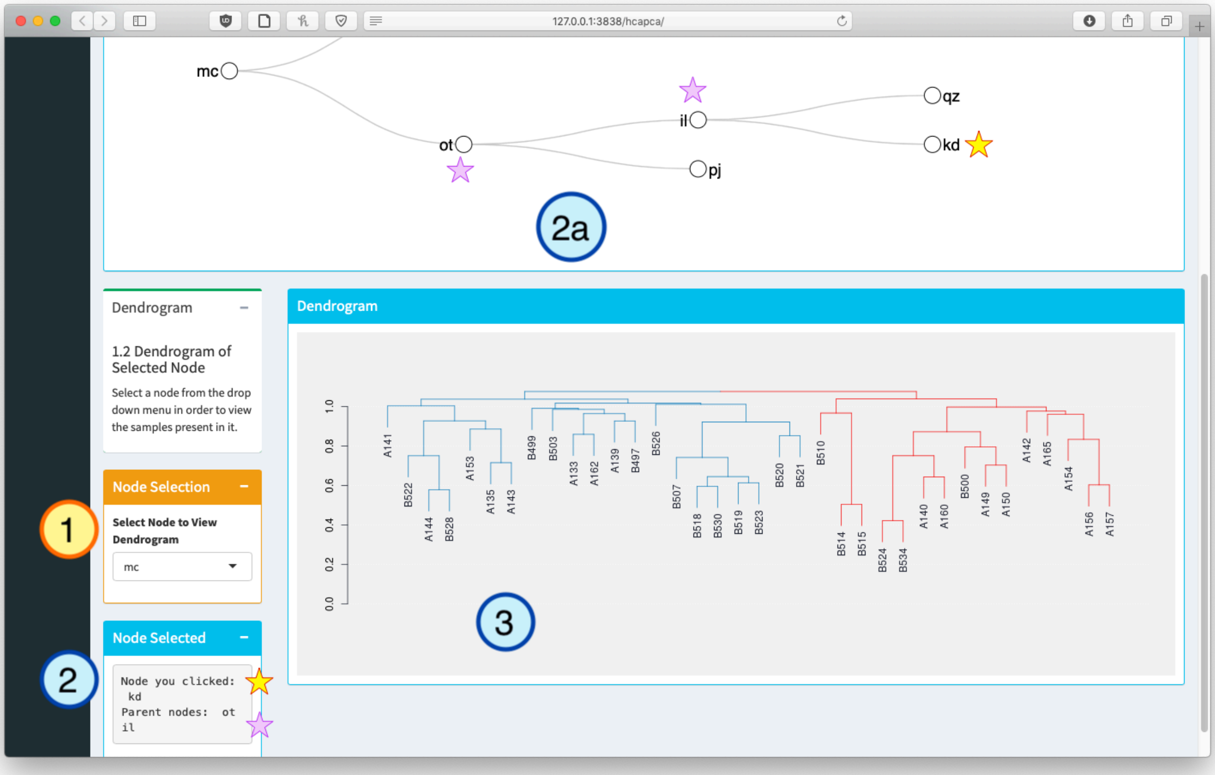

Figure S6. *The user can select which a node (1) from a drop-down menu and view its dendrogram (3). The blue box labelled "Node Selected" (2) shows the parent nodes of the node you click on at the top (2a)– in this case, "ot" and "il" are the parent nodes of "kd", t the node clicked on (indicated by colored stars).*

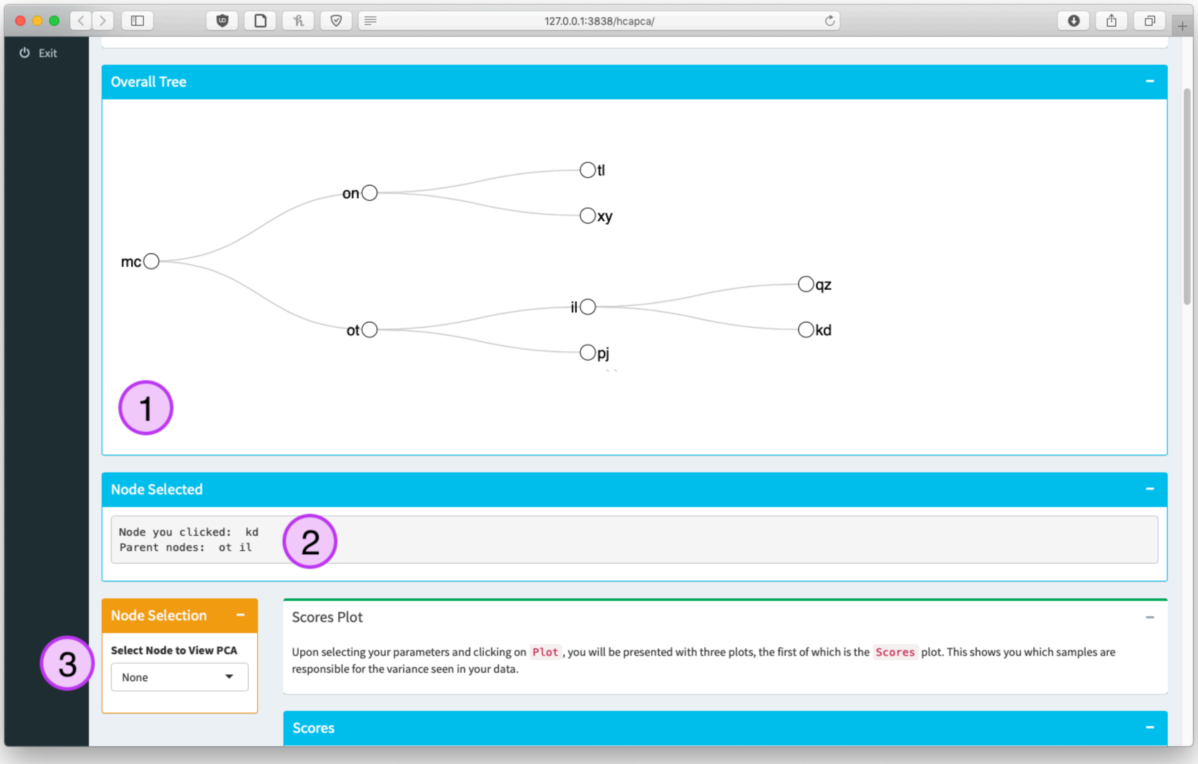

Figure S7. *The "PCA" tab of the results showing the overall tree (1). Clicking on a node shows the node as well as its parents (2) as explained earlier. The user can select a node to display from a dropdown menu (3).*

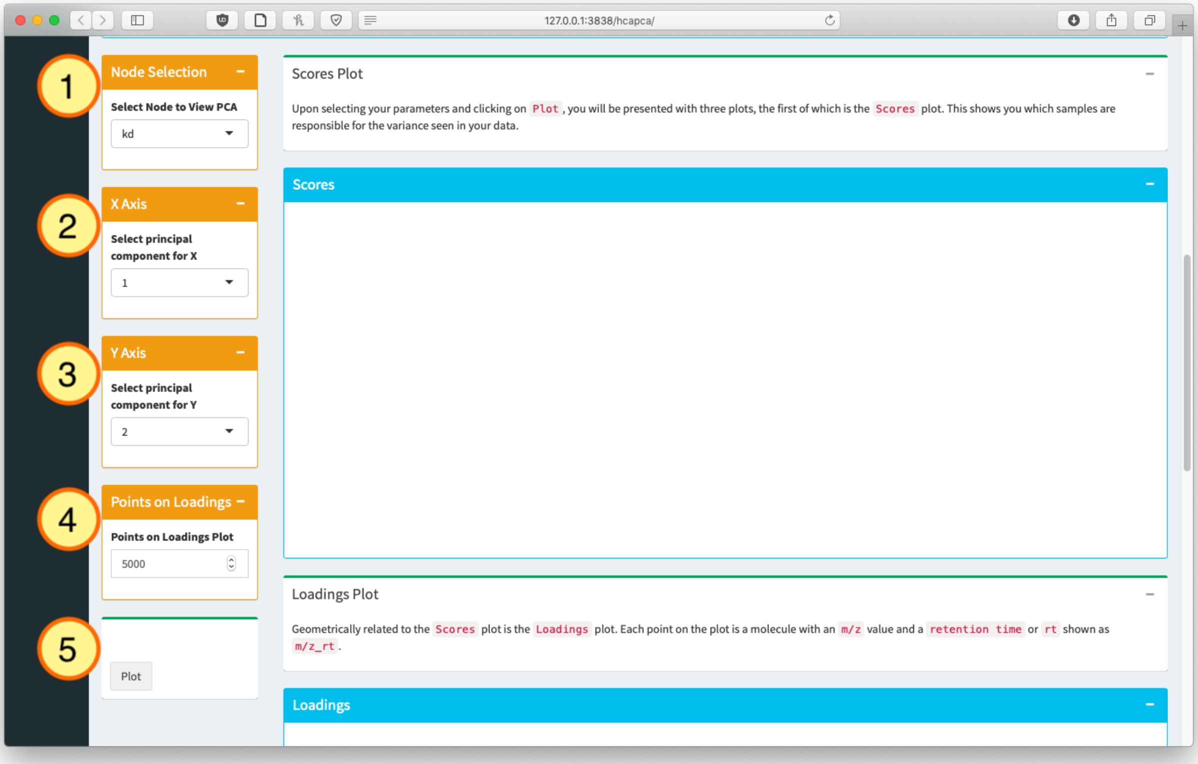

Figure S8. *The "PCA" tab of the results. The orange boxes on the left allow the user to select which node to plot (1), select the principal component for the X axis (2), select the principal component for the Y axis (3), select how many points they want shown on the loadings plot up to a maximum of 10,000 (4), and finally click the button labeled "Plot" to show the plots (5)*

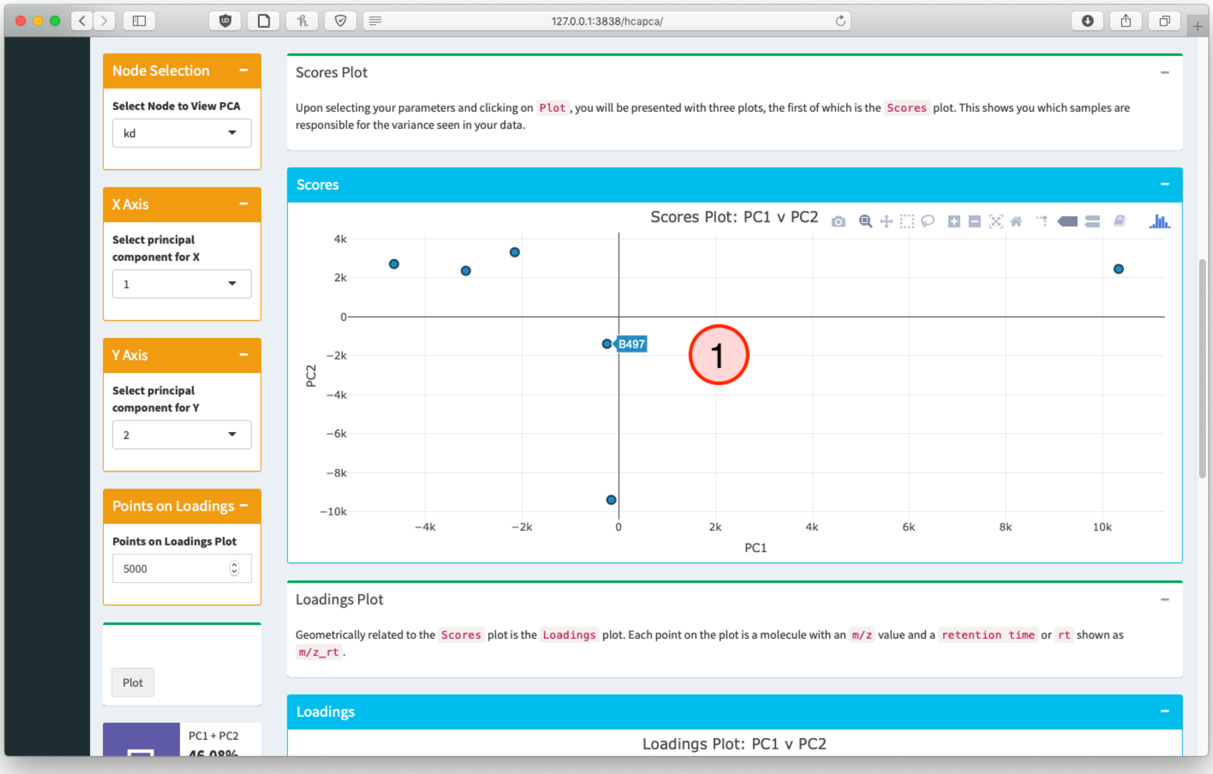

Figure S9. *The Scores plot is drawn with blue points (1). Hovering over a point with the mouse shows a popup of the sample name.*

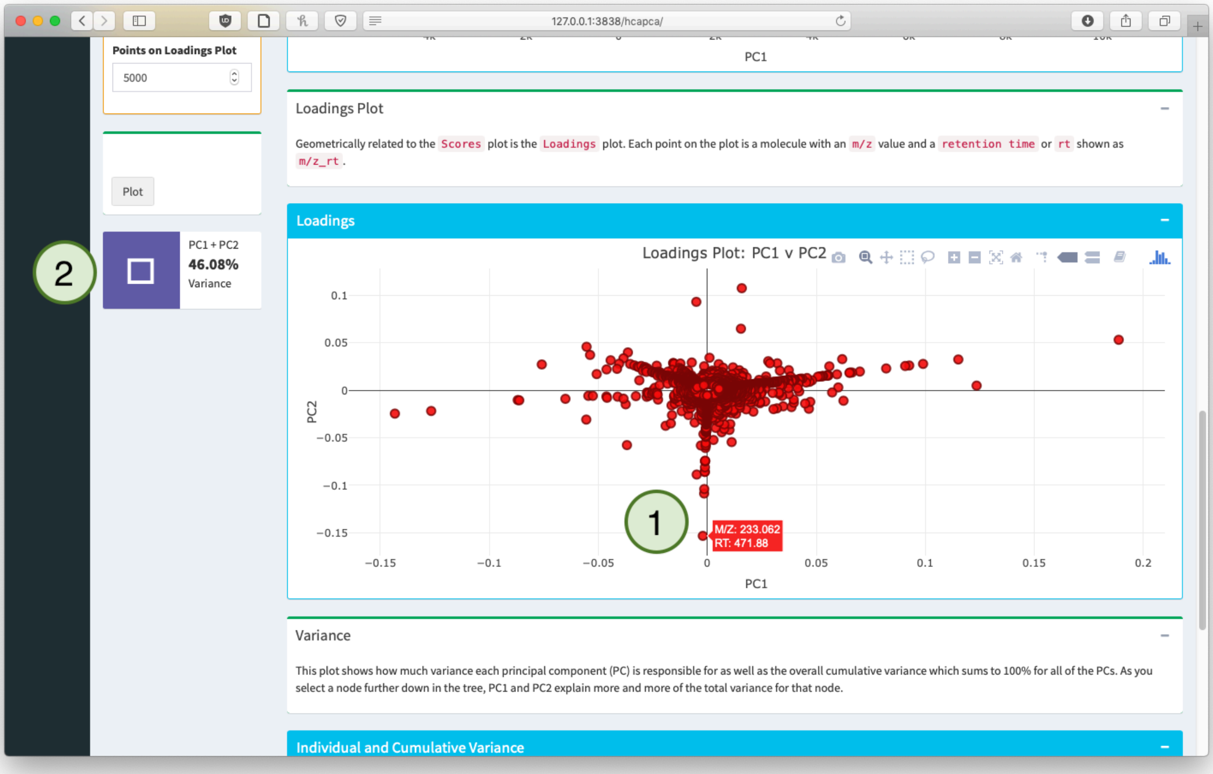

Figure S10. *The Loadings plot is drawn with red points (1). Hovering over a point with the mouse shows a popup of the mass-to-charge ratio (MZ) and retention time (RT). The variance explained by PC1 and PC2 is written in the small purple box on the left (2).*

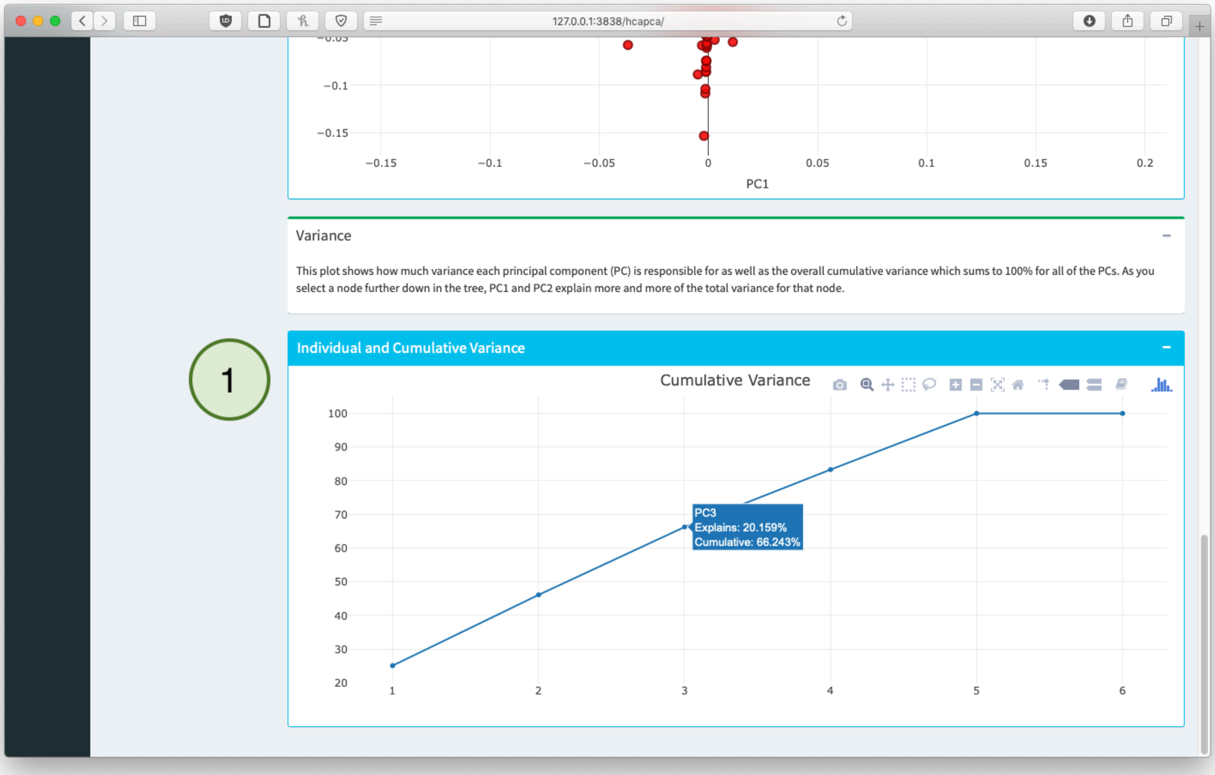

Figure S11. *The Plot showing individual and cumulative variance for all of the principal components (PCs) in the currently drawn PCA are shown (1). Hovering over a point with the mouse shows a popup with the name of the principal component, the variance explained by that component and the cumulative sum of the variances from 1 to that PC.*

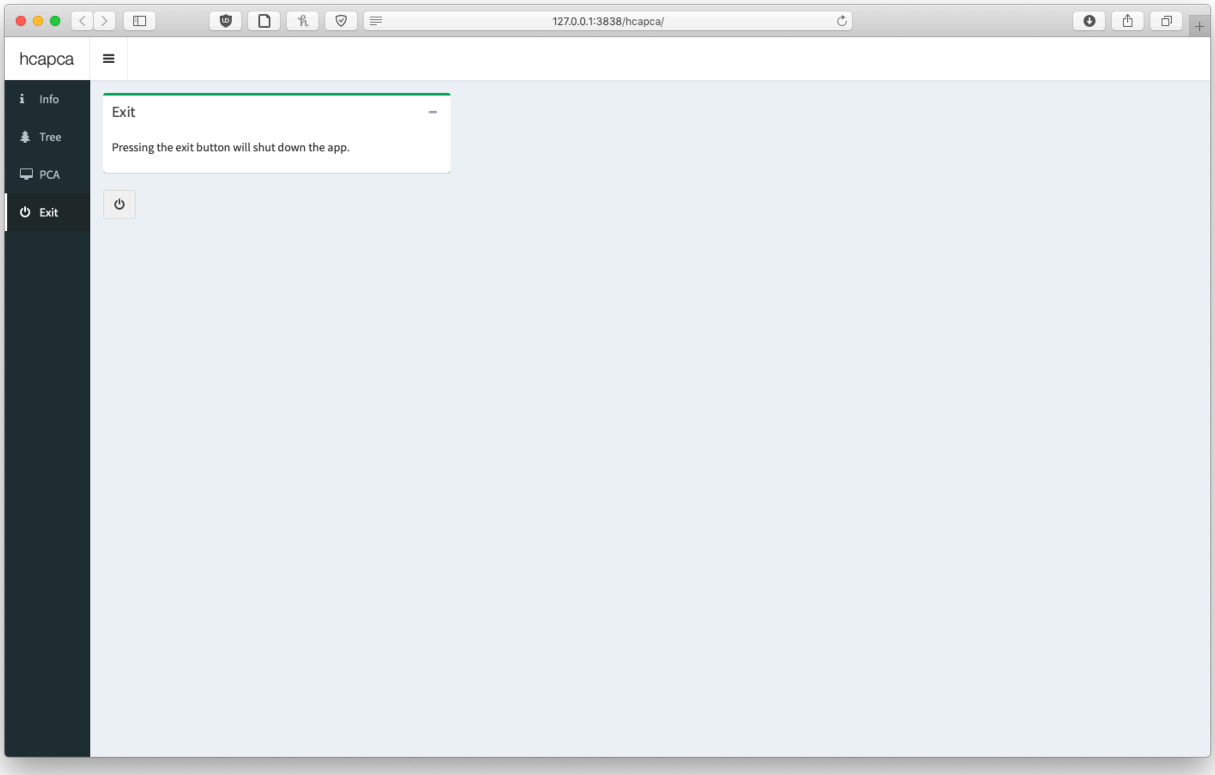

Figure S12. *The "Exit" tab of the results. Clicking the power button will exit the application. The user may also just close the internet browser tab / window to exit.*

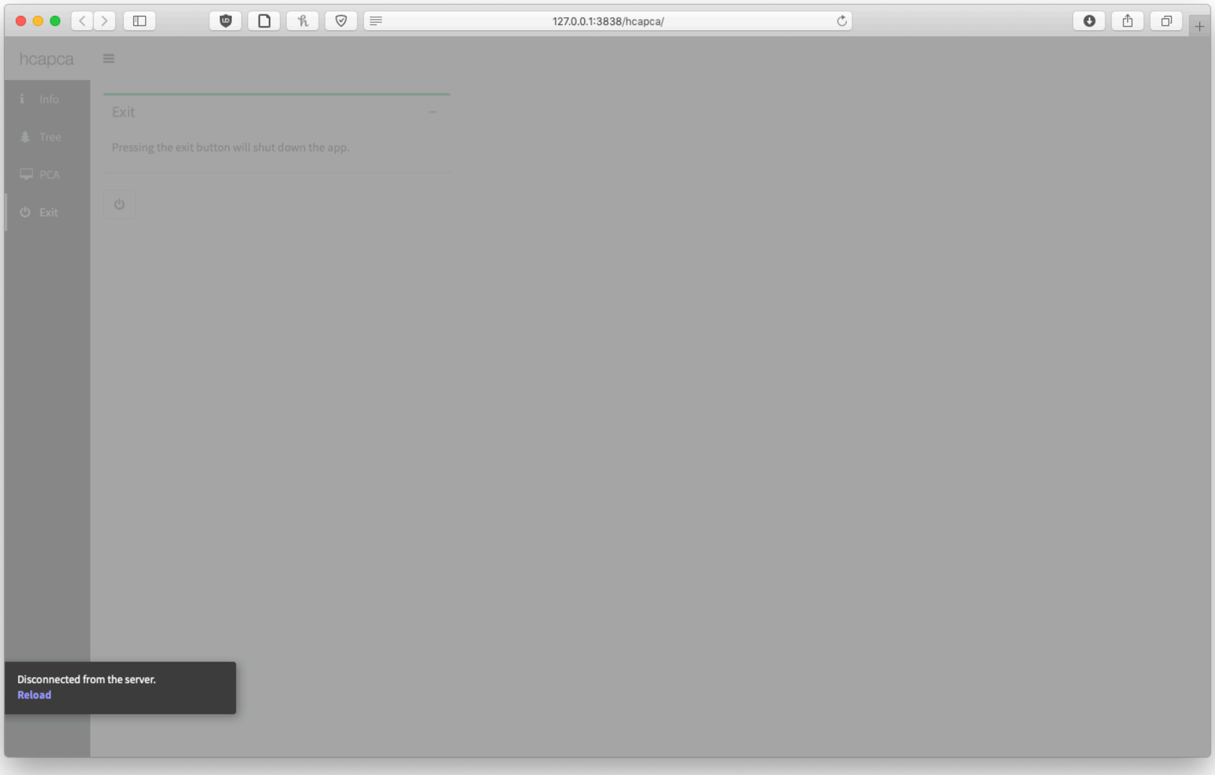

Figure S13. *Upon clicking the exit button, the application is greyed out. If the user chooses to close the tab/window, this would not be displayed since the tab/window itself would not exist.*

Table S1. Summary of Media compositions relevant to microbial strain fermentations.

Media	Recipe (all ingredients in g/L unless otherwise specified)
**ASW-A**	Soluble starch – 20<br>Glucose – 10<br>Peptone – 5<br>Yeast extract – 5<br>Calcium carbonate ($CaCO_3$) – 5<br>Artificial seawater – 1L
**ISP2**	Yeast extract – 4<br>Malt extract – 10<br>Dextrose – 4<br>Agar – 15<br>Artificial seawater – 1L
**ISP3**	Oatmeal – 20<br>Agar – 15<br>Artificial seawater – 1L
**R2A**	Yeast extract – 0.5<br>Peptone – 0.5<br>Casamino acids – 0.5<br>Dextrose – 0.5<br>Soluble Starch – 0.5<br>Sodium Pyruvate – 0.3<br>Dipotassium Phosphate – 0.3<br>Magnesium Sulphate – 0.05<br>Agar – 15<br>Artificial seawater – 1L
**R2A+DesI**	R2A medium with desferrioxamine added at 0.1mg/ml and Ferrous Sulfate ($FeSO_4$) at equimolar ratio (Desferrioxamine was purified from laboratory grown micromonospora)
**Gauze1**	Starch – 20<br>Potassium Nitrate (KNO_3) – 1<br>Dihydrogen Phosphate (H_2PO_4) – 0.5<br>Ferrous Sulfate Heptahydrate ($FeSO_4.7H_2O$) – 0.01<br>Agar – 15<br>Artificial seawater – 1L
**HV**	Humic Acid (dissolved in 10 mL 0.2N NaOH) – 1<br>Sodium Phosphate dibasic (Na_2HPO_4) – 0.5<br>Potassium Chloride (KCl) – 1.71<br>Magnesium Sulfate Heptahydrate ($MgSO_4.7H_2O$) – 0.05<br>Ferrous Sulfate Heptahydrate ($FeSO_4.7H_2O$) – 0.01<br>Calcium Carbonate (CaCO3) – 0.02<br>Yeast extract – 0.5<br>Agar – 15<br>Artificial seawater – 1L
**M4**	L-asparagine – 0.1<br>Potassium Phosphate Dibasic (K_2HPO_4) – 0.5<br>Ferrous Sulfate ($FeSO_4$) – 0.001<br>Magnesium Sulfate ($MgSO_4$) – 0.1<br>Peptone – 2

	Sodium Propionate – 4<br>Sodium Chloride (NaCl) – 20<br>Agar - 15
**Bonito**	Ground Bonito flakes - 2<br>Glucose – 2<br>Peptone – 2<br>Monopotassium Phosphate (KH_2PO_4) – 1<br>Ammonium Chloride (NH_4Cl) – 1<br>Agar – 15<br>Artificial seawater – 1L

The authors are grateful to a number of R package developers. The citations of these packages are listed below:

1. Therneau, T.M. A Package for Survival Analysis in R; 2020;
2. Ooms, J. askpass: Safe Password Entry for R, Git, and SSH; 2019;
3. Wickham, H. assertthat: Easy Pre and Post Assertions; 2019;
4. Lang, M.; R Core Team backports: Reimplementations of Functions Introduced Since R-3.0.0; 2020;
5. Urbanek, S. base64enc: Tools for base64 encoding; 2015;
6. Eddelbuettel, D.; Emerson, J.W.; Kane, M.J. BH: Boost C++ Header Files; 2020;
7. Francois, R. bibtex: Bibtex Parser; 2020;
8. Canty, A.; Ripley, B.D. boot: Bootstrap R (S-Plus) Functions; 2019;
9. Davison, A.C.; Hinkley, D.V. Bootstrap Methods and Their Applications; Cambridge University Press: Cambridge, 1997;
10. Csðrdi, G.; Chang, W. callr: Call R from R; 2020;
11. Csðrdi, G. cli: Helpers for Developing Command Line Interfaces; 2020;
12. Maechler, M.; Rousseeuw, P.; Struyf, A.; Hubert, M.; Hornik, K. cluster: Cluster Analysis Basics and Extensions; 2019;
13. Tierney, L. codetools: Code Analysis Tools for R; 2018;
14. Khan, A. collapsibleTree: Interactive Collapsible Tree Diagrams using "D3.js"; 2018;
15. Zeileis, A.; Fisher, J.C.; Hornik, K.; Ihaka, R.; McWhite, C.D.; Murrell, P.; Stauffer, R.; Wilke, C.O. colorspace: A Toolbox for Manipulating and Assessing Colors and Palettes; arXiv.org E-Print Archive, 2019;
16. Allaire, J.J. config: Manage Environment Specific Configuration Values; 2018;
17. Csðrdi, G. crayon: Colored Terminal Output; 2017;
18. Cheng, J. crosstalk: Inter-Widget Interactivity for HTML Widgets; 2020;
19. Ooms, J. curl: A Modern and Flexible Web Client for R; 2019;
20. Dowle, M.; Srinivasan, A. data.table: Extension of `data.frame`; 2019;
21. Glur, C. data.tree: General Purpose Hierarchical Data Structure; 2019;
22. Galili, T. dendextend: an R package for visualizing, adjusting, and comparing trees of hierarchical clustering. Bioinformatics 2015, doi:10.1093/bioinformatics/btv428.
23. Csðrdi, G.; Mðller, K.; Hester, J. desc: Manipulate DESCRIPTION Files; 2018;
24. Lucas, D.E. with contributions by A.; Tuszynski, J.; Bengtsson, H.; Urbanek, S.; Frasca, M.; Lewis, B.; Stokely, M.; Muehleisen, H.; Murdoch, D.; Hester, J.; et al. digest: Create Compact Hash Digests of R Objects; 2020;
25. Wickham, H.; Franðois, R.; Henry, L.; Mðller, K. dplyr: A Grammar of Data Manipulation; 2020;

26. Xie, Y.; Cheng, J.; Tan, X. DT: A Wrapper of the JavaScript Library "DataTables"; 2020;
27. Wickham, H. ellipsis: Tools for Working with ...; 2019;
28. Zeileis, A.; Hornik, K.; Murrell, P. Escaping RGBland: Selecting Colors for Statistical Graphics. Computational Statistics & Data Analysis 2009, 53, 3259–3270, doi:10.1016/j.csda.2008.11.033.
29. Wickham, H.; Xie, Y. evaluate: Parsing and Evaluation Tools that Provide More Details than the Default; 2019;
30. Eddelbuettel, D.; Balamuta, J.J. Extending extitR with extitC++: A Brief Introduction to extitRcpp. PeerJ Preprints 2017, 5, e3188v1, doi:10.7287/peerj.preprints.3188v1.
31. Gaslam, B. fansi: ANSI Control Sequence Aware String Functions; 2020;
32. Pedersen, T.L.; Nicolae, B.; Franðois, R. farver: High Performance Colour Space Manipulation; 2020;
33. Wood, S.N. Fast stable restricted maximum likelihood and marginal likelihood estimation of semiparametric generalized linear models. Journal of the Royal Statistical Society (B) 2011, 73, 3–36.
34. Chang, W. fastmap: Fast Implementation of a Key-Value Store; 2019;
35. R Core Team foreign: Read Data Stored by "Minitab", "S", "SAS", "SPSS", "Stata", "Systat", "Weka", "dBase", ...; 2020;
36. Wood, S.N. Generalized Additive Models: An Introduction with R; 2nd ed.; Chapman and Hall/CRC, 2017;
37. Wickham, H. ggplot2: Elegant Graphics for Data Analysis; Springer-Verlag New York, 2016; ISBN 978-3-319-24277-4.
38. Hester, J. glue: Interpreted String Literals; 2020;
39. Auguie, B. gridExtra: Miscellaneous Functions for "Grid" Graphics; 2017;
40. Wickham, H.; Pedersen, T.L. gtable: Arrange "Grobs" in Tables; 2019;
41. Carr, D.; Lewin-Koh, ported by N.; Maechler, M.; Sarkar, contains copies of lattice functions written by D. hexbin: Hexagonal Binning Routines; 2020;
42. RStudio; Inc. htmltools: Tools for HTML; 2019;
43. Vaidyanathan, R.; Xie, Y.; Allaire, J.J.; Cheng, J.; Russell, K. htmlwidgets: HTML Widgets for R; 2019;
44. Cheng, J.; Bravo, H.C.; Ooms, J.; Chang, W. httpuv: HTTP and WebSocket Server Library; 2019;
45. Wickham, H. httr: Tools for Working with URLs and HTTP; 2019;
46. Sievert, C. Interactive Web-Based Data Visualization with R, plotly, and shiny; Chapman and Hall/CRC, 2020; ISBN 978-1-138-33145-7.
47. Wilke, C.O. isoband: Generate Isolines and Isobands from Regularly Spaced Elevation Grids; 2020;
48. Wand, M. KernSmooth: Functions for Kernel Smoothing Supporting Wand & Jones (1995); 2019;
49. Talbot, J. labeling: Axis Labeling; 2014;
50. Cheng, J.; Chang, W. later: Utilities for Scheduling Functions to Execute Later with Event Loops; 2019;
51. Sarkar, D. Lattice: Multivariate Data Visualization with R; Springer: New York, 2008;
52. Wickham, H. lazyeval: Lazy (Non-Standard) Evaluation; 2019;
53. Henry, L. lifecycle: Manage the Life Cycle of your Package Functions; 2020;
54. Bache, S.M.; Wickham, H. magrittr: A Forward-Pipe Operator for R; 2014;
55. Bates, D.; Maechler, M. Matrix: Sparse and Dense Matrix Classes and Methods; 2019;
56. Xie, Y. mime: Map Filenames to MIME Types; 2020;

57. Terry M. Therneau; Patricia M. Grambsch Modeling Survival Data: Extending the Cox Model; Springer: New York, 2000; ISBN 0-387-98784-3.
58. Venables, W.N.; Ripley, B.D. Modern Applied Statistics with S; Fourth.; Springer: New York, 2002;
59. Wickham, C. munsell: Utilities for Using Munsell Colours; 2018;
60. Pinheiro, J.; Bates, D.; DebRoy, S.; Sarkar, D.; R Core Team nlme: Linear and Nonlinear Mixed Effects Models; 2020;
61. Ooms, J. openssl: Toolkit for Encryption, Signatures and Certificates Based on OpenSSL; 2019;
62. Mðller, K.; Wickham, H. pillar: Coloured Formatting for Columns; 2020;
63. Wickham, H.; Hester, J. pkgbuild: Find Tools Needed to Build R Packages; 2020;
64. Csðrdi, G. pkgconfig: Private Configuration for "R" Packages; 2019;
65. Wickham, H.; Hester, J.; Chang, W. pkgload: Simulate Package Installation and Attach; 2018;
66. Mðller, K. plogr: The "plog" C++ Logging Library; 2018;
67. Csðrdi, G.; Sorhus, S. praise: Praise Users; 2015;
68. Csðrdi, G. prettyunits: Pretty, Human Readable Formatting of Quantities; 2020;
69. Csðrdi, G.; Chang, W. processx: Execute and Control System Processes; 2020;
70. Cheng, J. promises: Abstractions for Promise-Based Asynchronous Programming; 2019; Loden, J.; Daeschler, D.; Rodola', G.; Csðrdi, G. ps: List, Query, Manipulate System Processes; 2020;
71. Henry, L.; Wickham, H. purrr: Functional Programming Tools; 2020;
72. Gagolewski, M. R package stringi: Character string processing facilities; 2020;
73. R Core Team R: A Language and Environment for Statistical Computing; R Foundation for Statistical Computing: Vienna, Austria, 2020;
74. Chang, W. R6: Encapsulated Classes with Reference Semantics; 2019;
75. Neuwirth, E. RColorBrewer: ColorBrewer Palettes; 2014;
76. Eddelbuettel, D.; François, R. Rcpp: Seamless R and C++ Integration. Journal of Statistical Software 2011, 40, 1–18, doi:10.18637/jss.v040.i08.
77. Henry, L.; Wickham, H. rlang: Functions for Base Types and Core R and "Tidyverse" Features; 2020;
78. Therneau, T.; Atkinson, B. rpart: Recursive Partitioning and Regression Trees; 2019;
79. Mðller, K. rprojroot: Finding Files in Project Subdirectories; 2018;
80. Ushey, K.; Allaire, J.J.; Wickham, H.; Ritchie, G. rstudioapi: Safely Access the RStudio API; 2020;
81. Wickham, H.; Seidel, D. scales: Scale Functions for Visualization; 2020;
82. Eddelbuettel, D. Seamless R and C++ Integration with Rcpp; Springer: New York, 2013;
83. Chang, W.; Cheng, J.; Allaire, J.J.; Xie, Y.; McPherson, J. shiny: Web Application Framework for R; 2020;
84. Chang, W.; Ribeiro, B.B. shinydashboard: Create Dashboards with "Shiny"; 2018;
85. Wood, S.N.; N.; Pya; S"afken, B. Smoothing parameter and model selection for general smooth models (with discussion). Journal of the American Statistical Association 2016, 111, 1548–1575.
86. Stauffer, R.; Mayr, G.J.; Dabernig, M.; Zeileis, A. Somewhere over the Rainbow: How to Make Effective Use of Colors in Meteorological Visualizations. Bulletin of the American Meteorological Society 2009, 96, 203–216, doi:10.1175/BAMS-D-13-00155.1.
87. Ushey, K. sourcetools: Tools for Reading, Tokenizing and Parsing R Code; 2018;
88. Wood, S.N. Stable and efficient multiple smoothing parameter estimation for generalized additive models. Journal of the American Statistical Association 2004, 99, 673–686.

89. Wickham, H. stringr: Simple, Consistent Wrappers for Common String Operations; 2019;
90. Ooms, J. sys: Powerful and Reliable Tools for Running System Commands in R; 2019;
91. Wickham, H. testthat: Get Started with Testing. The R Journal 2011, 3, 5–10.
92. Ooms, J. The jsonlite Package: A Practical and Consistent Mapping Between JSON Data and R Objects. arXiv:1403.2805 [stat.CO] 2014.
93. Wood, S.N. Thin-plate regression splines. Journal of the Royal Statistical Society (B) 2003, 65, 95–114.
94. Mðller, K.; Wickham, H. tibble: Simple Data Frames; 2020;
95. Wickham, H.; Henry, L. tidyr: Tidy Messy Data; 2020;
96. Henry, L.; Wickham, H. tidyselect: Select from a Set of Strings; 2020;
97. Team, R.C.; worldwide, contributors translations: The R Translations Package;
98. Perry, P.O. utf8: Unicode Text Processing; 2018;
99. Wickham, H.; Henry, L.; Vaughan, D. vctrs: Vector Helpers; 2020;
100. Garnier, S. viridis: Default Color Maps from “matplotlib”; 2018;
101. Garnier, S. viridisLite: Default Color Maps from “matplotlib” (Lite Version); 2018;
102. Hester, J.; Mðller, K.; Ushey, K.; Wickham, H.; Chang, W. withr: Run Code “With” Temporarily Modified Global State; 2020;
103. Dahl, D.B.; Scott, D.; Roosen, C.; Magnusson, A.; Swinton, J. xtable: Export Tables to LaTeX or HTML; 2019;
104. Stephens, J.; Simonov, K.; Xie, Y.; Dong, Z.; Wickham, H.; Horner, J.; reikoch; Beasley, W.; O’Connor, B.; Warnes, G.R. yaml: Methods to Convert R Data to YAML and Back; 2020;
